# PW02-034 - NLRP3 mosaicism detection in CAPS using NGS

**DOI:** 10.1186/1546-0096-11-S1-A175

**Published:** 2013-11-08

**Authors:** M Stoffels, M Elferink, P van Zon, J Frenkel, E Hoppenreijs, A Simon, M van Gijn

**Affiliations:** 1 Department of General Internal Medicine, Radboud University Nijmegen Medical Centre, Netherlands; 2 Nijmegen Centre for Infection, Inflammation and Immunity (N4i), Netherlands; 3 Nijmegen Centre for Molecular Life Sciences, Nijmegen, Netherlands; 4 Department of Medical Genetics, Netherlands; 5 Department of Pediatrics, UMC Utrecht, Utrecht, Netherlands; 6 Department of Pediatric Rheumatology, Radboud University Nijmegen Medical Centre, Nijmegen, Netherlands

## Introduction

Heterozygous germline mutations in *NLRP3* are a known cause of Cryopyrin associated periodic syndrome (CAPS). However, in a considerable number of these patients mutations cannot be detected by conventional genetic analyses. Somatic mosaicism has been detected in several mutation-negative patients, and is suggested to be a major cause of CAPS in these patients.

## Objectives

Using next-generation sequencing (NGS), we aimed to investigate whether mosaicism for *NLRP3* mutations was present in mutation-negative CAPS patients.

## Methods

Six well-defined mutation-negative CAPS patients were included. In addition two CAPS patients that were identified before as mosaics, by a subcloning and Sanger sequencing method, were included for validation purposes. In short, barcoded whole genome fragment libraries were generated for each patient, enriched for the coding regions of 300 inflammation related genes using a custom Agilent 1M microarray and subsequently sequenced on the SOLiD5500XL platform. Because almost all *NLRP3* mutations are located in exon 3 of *NLRP3*, this exon was analyzed using an in house bioinformatic pipeline, CARTAGENIA BENCH lab NGS and IGV2.2 software.

## Results

In all patients all *NLRP3* exons were 100% covered, with an average coverage of 460x. The two patients that were identified before as being 6.3% mosaic for the E567K and G755R mutation demonstrated mosaicism for these mutations of subsequently approximately 8% and 10%. This indicated the method was valid to study mosaicism. We could not detect mosaicism for known pathogenic mutations in exon 3 in the six patients. However, in one patient 9% mosaicism for an E567G variant was detected. Although this variant has not been described as heterozygous mutation in CAPS patients, mosaicism for this variant has been described before in a mutation-negative CAPS patient. The clinical relevance of this variant, however, remains uncertain.

## Conclusion

We demonstrated that our NGS method is a reliable, accurate method to identify mosaic percentages of at least 8-10%. In contrast to the earlier reported finding that in 70 percent of mutation-negative CAPS patients mosaicism for *NLRP3* mutations can be demonstrated, we could not find evidence for mosaicism in exon 3 of *NLRP3* in five of our six patients. Although very low mosaic percentages or mosaic mutations in other exons cannot be excluded, this suggests that next to germline and mosaic *NLRP3* mutations also other, not yet identified defects underlie CAPS.

## Competing interests

None declared

